# Immunization with Hexon Modified Adenoviral Vectors Integrated with gp83 Epitope Provides Protection against *Trypanosoma cruzi* Infection

**DOI:** 10.1371/journal.pntd.0003089

**Published:** 2014-08-21

**Authors:** Anitra L. Farrow, Girish Rachakonda, Linlin Gu, Valentina Krendelchtchikova, Pius N. Nde, Siddharth Pratap, Maria F. Lima, Fernando Villalta, Qiana L. Matthews

**Affiliations:** 1 Department of Medicine, Division of Infectious Diseases, University of Alabama at Birmingham, Birmingham, Alabama, United States of America; 2 Department of Microbiology and Immunology, School of Medicine, Meharry Medical College, Nashville, Tennessee, United States of America; 3 Center for AIDS Research, University of Alabama at Birmingham, Birmingham, Alabama, United States of America; Federal University of São Paulo, Brazil

## Abstract

**Background:**

*Trypanosoma cruzi* is the causative agent of Chagas disease. Chagas disease is an endemic infection that affects over 8 million people throughout Latin America and now has become a global challenge. The current pharmacological treatment of patients is unsuccessful in most cases, highly toxic, and no vaccines are available. The results of inadequate treatment could lead to heart failure resulting in death. Therefore, a vaccine that elicits neutralizing antibodies mediated by cell-mediated immune responses and protection against Chagas disease is necessary.

**Methodology/Principal Findings:**

The “antigen capsid-incorporation” strategy is based upon the display of the *T. cruzi* epitope as an integral component of the adenovirus' capsid rather than an encoded transgene. This strategy is predicted to induce a robust humoral immune response to the presented antigen, similar to the response provoked by native Ad capsid proteins. The antigen chosen was *T. cruzi* gp83, a ligand that is used by *T. cruzi* to attach to host cells to initiate infection. The gp83 epitope, recognized by the neutralizing MAb 4A4, along with His_6_ were incorporated into the Ad serotype 5 (Ad5) vector to generate the vector Ad5-HVR1-gp83-18 (Ad5-gp83). This vector was evaluated by molecular and immunological analyses. Vectors were injected to elicit immune responses against gp83 in mouse models. Our findings indicate that mice immunized with the vector Ad5-gp83 and challenged with a lethal dose of *T. cruzi* trypomastigotes confer strong immunoprotection with significant reduction in parasitemia levels, increased survival rate and induction of neutralizing antibodies.

**Conclusions/Significance:**

This data demonstrates that immunization with adenovirus containing capsid-incorporated *T. cruzi* antigen elicits a significant anti-gp83-specific response in two different mouse models, and protection against *T. cruzi* infection by eliciting neutralizing antibodies mediated by cell-mediated immune responses, as evidenced by the production of several Ig isotypes. Taken together, these novel results show that the recombinant Ad5 presenting *T. cruzi* gp83 antigen is a useful candidate for the development of a vaccine against Chagas disease.

## Author Summary

Chagas disease is caused by the protozoan parasite *Trypanosoma cruzi* and has been detrimental to millions of people in Latin America since the early 1900s, and now this disease is having a global impact. Decades later, there is still no vaccine for this disease. Scientists have used the adenovirus vector in gene therapy as well as vaccine therapy for many diseases. In this study, adenovirus is used as the host vector for a potential vaccine against Chagas disease in a non-traditional way. Instead of the antigen being expressed as a transgene product, the antigen is incorporated on the capsid of the adenovirus to evoke an enhanced humoral immune response against *T. cruzi*. This study highlights the modified adenovirus' ability to elicit a strong anti-*T. cruzi* humoral response as well as providing protection against *Trypanosoma cruzi* infection. These findings illustrate the true potential of generating an effective *T. cruzi* vaccine by means of unique gene therapy-based techniques.

## Introduction

*Trypanosoma cruzi* is the intracellular parasite that causes Chagas disease [1]. Chagas disease is an acute and chronic infection that affects over 8 million people throughout endemic regions in Mexico, Central and South America [2]. Recently, the disease has received increasing attention as an emerging health problem in North America, Europe, Japan and Australia due to international migrations from endemic areas to non-endemic areas [3], [4], posing a new significant worldwide health challenge. Over the decades, there has been an increase in the number of infected persons residing in the United States with over 300,000 reported cases [5]. *T. cruzi* is transmitted to the mammalian host at the site of a triatomine bug bite [6]. Nifurtimox and Benznidazol are current treatments for this infection [7]. These anti-parasitic drugs are 80% successful in curing the acute phase with severe side effects, but ineffective in curing the chronic phase. The results of inadequate treatment could lead to severe arrhythmias, congestive heart failure, and sudden death [8], [9], common pathological features of the disease. There is no vaccine against Chagas disease at present and progress has been slow despite several years of effort. Therefore, an improved treatment, such as a vaccine against Chagas disease is necessary.

Human adenovirus type 5 (Ad5) has been used as a vehicle for many preclinical and clinical vaccines such as Ebola, malaria, simian immunodeficiency virus (SIV), and human immunodeficiency virus (HIV) [10]–[13]. One advantage of using adenoviral vectors in gene therapy and vaccine approaches is the vectors' ability to efficiently infect a wide variety of cell types. Ad5 vectors also have the capability to contain DNA inserts up to 8 kilobases [14]–[17] and can transduce their genome in replicating and nonproliferating cells. Lastly, adenoviral vectors are easily manipulated using recombinant DNA techniques [18]. We sought out to utilize Ad5 and the “antigen capsid-incorporation” strategy [19] to design a novel vaccine approach against Chagas disease. This strategy consists of incorporating antigenic peptides within the capsid structure of the adenoviral vectors rather than an encoded transgene [16]. The capsid-incorporated antigen strategy can induce a robust humoral immune response to the presented antigen, similar to the response provoked by native Ad capsid proteins [20]–[22].

In one of our first publications, we utilized the antigen capsid-incorporation strategy and modified Ad5 to contain a seven amino acid region of the HIV glycoprotein 41. This incorporation was displayed within the hexon hypervariable region 2 of the virus capsid. We were able to generate a HIV epitope-specific humoral response through prime-boost immunization in BALB/c mice [16]. The goal of our current study was to use our incorporation strategy and design a recombinant Ad5 vector that presents a *T. cruzi* antigen to elicit epitope-specific humoral responses. Glycoprotein 83 (gp83), is a trans-sialidase like molecule used by *T. cruzi* trypomastigotes as a ligand to attach to host cells and initiate infection [23]. We have shown that the gp83 is expressed only in invasive trypomastigotes [24] and that blocking *T. cruzi* gp83 with MAb 4A4, that recognizes a conformational epitope on gp83, neutralizes trypomastigote cellular infection [24]. We also have shown that passive immunization with monovalent Fab fragments of the MAb 4A4 neutralizes *T. cruzi* infection in rodent models of Chagas disease challenged with a lethal dose of trypomastigotes [23]. Since passive immunization with neutralizing MAb 4A4 confers significant immune protection in mice infected with *T. cruzi* in terms of significant reduction of parasitemia and increased mice survival rate with respect to controls [23], we reasoned that a vaccine that induces neutralizing antibodies would provide immune protection. Therefore, to test this hypothesis we have chosen to incorporate the gp83 epitope recognized by the neutralizing MAb 4A4 [24] within the Ad5 major capsid protein hexon to evaluate whether this vaccine elicits an immune protective response. We provide evidence that the *T. cruzi* gp83 antigen was incorporated into the hypervariable region 1 (HVR1) of Ad5. We confirmed through mouse immunizations that the recombinant anti-*T. cruzi* vectors could elicit epitope-specific humoral immune responses in two different strains of mice. We also confirmed that upon challenge with trypomastigotes, the mice immunized with the antigen capsid-incorporated vector displayed a significant reduction in parasitemia, improvement in their survival rate and were able to elicit neutralizing antibodies.

## Materials and Methods

### Antibodies

Anti-His_6_ mouse monoclonal antibody (MAb) was purchased from GenScript (Piscataway, NJ). Anti-Penta-His MAb was purchased from Qiagen (Valencia, CA). Horseradish peroxidase (HRP)-conjugated goat anti-mouse secondary antibody was purchased from Millipore (Temecula, CA). Isotype-specific goat anti-mouse antibodies were purchased from Sigma-Aldrich (St. Louis, MO). Adenovirus fiber antibody (4D2) was purchased from Abcam (Cambridge, MA). Donkey anti-goat HRP-conjugated antibody was purchased from Jackson ImmunoResearch Laboratories, Inc. (West Grove, PA).

### Cell culture and parasites

Human embryonic kidney (HEK293) cells were obtained from and cultured in the medium recommended by the American Type Culture Collection (Manassas, VA). The cell line was incubated at 37°C and 5% CO_2_ under humidified conditions.

The Tulahuen strain of *T. cruzi* [25] was used. Blood trypomastigotes used for challenging immunized mice were obtained from infected mice as described [26]. *T. cruzi* trypomastigotes expressing green fluorescent protein (GFP) for cellular infection assays were generated as described [27].

### Recombinant adenoviral construction

In order to generate recombinant adenovirus with the *T. cruzi* gp83 epitope (gp83 epitope generated from GenBank accession #: AY513728.1) as well as His_6_ incorporation genetically incorporated within Ad5 hexon HVR1, the following was performed: the DNA sequence corresponding to the mouse MAb 4A4 binding region of gp83 and His_6_ (24 amino acids) was generated by GenScript (Piscataway, NJ) and subcloned into the HVR1 region (the gp83 sequence replaced amino acids 139 to 144) of the H5/pH5S plasmid [28]. The resulting plasmid HVR1-gp83-18 was digested with EcoRI and PmeI. These resulting fragments containing the homologous recombination region and the hexon gene were purified, and then recombined with a SwaI-digested Ad5 backbone vector that lacks the hexon gene, pAd5/ΔH5 [29].

### Rescue, purification and titration of recombinant Ad5 vector

To rescue the vector, the replicative-defective recombinant adenoviral genome was digested with PacI and transfected with PolyJet (SignaGen Laboratories, Rockville, MD) into the Ad5-E1-expressing HEK293 cells. Multi-step large-scale propagations of recombinant Ad5 vector were performed after the vector was rescued. To purify the rescued vector, two-step cesium chloride ultracentrifugation was employed, followed by dialysis against 1× PBS containing 10% glycerol. To titrate the purified vectors, physical titers expressed as viral particles (VPs) per ml were measured using absorbance at 260 nm. The infectious particles (IPs) per ml were determined by TCID_50_ assay [30]. Modifications of the hexon gene were confirmed by PCR analysis with the primers pVI 1581 fwd: 5′-TATGTGTGTCATGTATGCGT-3′ and 3′HVR5:5′-GGCATGTAAGAAATATGAGTGTCTGGG-3′. To confirm the gp83 epitope-His_6_ incorporation within the hexon gene, PCR analysis was performed with the following primers: gp83-18Fwd: 5′-TATGTGTGTCATGTATGCGT-3′ and gp83-18Rev: 5′-TAGGTTGTGATGGTGATGGTGATG-3′.

### Western blot analysis

To analyze gp83 epitope and His_6_ expression, 5×10^9^ VPs/vector were boiled and resolved on SDS-PAGE gels, followed by transfer onto polyvinylidene difluoride (PVDF) membranes, which were then blocked with 5% dry non-fat milk in Phosphate Buffered Saline with Tween 20 (PBST) buffer (1× PBS and, 0.05% Tween 20) for 1 hour. The membrane was incubated overnight at 4°C with His_6_ MAb (1∶5,000 dilution in blocking buffer, GenScript, Piscataway, NJ), then washed, and incubated with HRP-conjugated goat anti-mouse antibody (1∶5,000; Millipore, Temecula, CA). The proteins were detected by using 3′3′-diaminobenzidine tablets (Sigma-Aldrich, MO) [30].

### Mice immunizations

BALB/C or C57BL/6 mice (6–8 weeks) were immunized with either Ad5 (control) or Ad5-gp83 to determine the gp83-specific immunogenicity. The groups of mice were intramuscularly immunized with the corresponding vector (1×10^10^ VP/mouse) at each time-point, with a two-week interval between prime, boost and reboost.

### Whole virus ELISAs, indirect ELISAs, and sera-based isotype ELISAs

In order to investigate the exposure-display of gp83 epitope and His_6_ on the surface of the capsid, whole virus ELISAs were performed as described elsewhere [28]. Briefly, different amounts of the Ad5-gp83 or Ad5 (control) were immobilized and blocked. The immobilized vector was incubated with His_6_ MAb (1∶2,000, GenScript, Piscataway, NJ), followed by incubation with the HRP-conjugated goat anti-mouse antibody (1∶5,000, Millipore, Temecula, CA). ELISAs were developed with the SIGMAFAST OPD peroxidase substrate (Sigma-Aldrich, MO) and measured at OD 450 nm.

Indirect ELISA was performed to detect gp83-specfic total IgG levels. ELISA plates were coated with 10 µM gp83-18 peptide (KIYWKQPVEGTKSWTLSK, GenScript, Piscataway, NJ) in 100 µl of 50 mM carbonate buffer per well as previously described in [16]. Unbound peptide was removed by washing with PBST buffer (1× PBS and, 0.05% Tween 20). The plate was then blocked with 5% non-fat milk/PBST for 1 hour at room temperature and 1 µl of serum samples (diluted 1∶100) from control and immunized mice were applied to the plate and incubated for 2 hours at room temperature. The plate was then extensively washed and blocked again followed by incubation with the HRP-conjugated goat anti-mouse antibody (1∶5,000, Millipore, Temecula, CA). ELISA was performed as described above. The amount of anti-gp83 antibodies in the sera was calculated based on a standard curve of mouse IgG protein.

Sera-based IgG isotype ELISAs were performed to determine the magnitude of gp83- specific humoral response and relative isotype development. Briefly, 10 µM gp83-18 peptide (KIYWKQPVEGTKSWTLSK) was used to coat the plates as described above. Immunized sera were diluted 100 fold, applied to the plates, and incubated for 2 hours. Then goat anti-mouse isotypes, IgG1, IgG2a and IgG2b (1∶1,000; Sigma-Aldrich, MO) were added, followed by incubation with HRP-conjugated donkey anti-goat antibody (1∶5,000, Jackson ImmunoResearch, PA). The ELISAs were developed as above.

### Mice challenge

Groups of five female C57BL/6 mice (Jackson Laboratory, 6 week-old) that were immunized with either Ad5 (control) or Ad5-gp83 as described above were challenged intraperitoneally with a lethal dose of 5×10^3^ blood trypomastigotes obtained from near the peak of parasitemia of C57BL/6 mice with the clone 20A of the Tulahuen strain of *T. cruzi*. Parasitemia was monitored by placing 5 µl of tail blood under a coverslip and counting 100 high-powered fields, as described elsewhere [31]. Animals reaching the peak of parasitemia were euthanized because they were moribund. IACUC regulations do not allow maintaining suffering moribund animals. This is consistent with federal policy and regulations for Public Health Service (PHS) funded grants to ensure the humane care and use of animals in research. We used a standardized model of infecting C57BL/6 mice of the same age, weight and sex with a lethal dose of highly infective trypomastigote clone 20A of the Tulahuen strain [25], which present similar levels of parasitemia including when reaching its peak. The day on which moribund mice were euthanized was recorded.

### Neutralization assay and fluorescent microscopy

To investigate whether vaccination of mice induce neutralizing antibodies, immunized or control mice were bled before challenging with *T. cruzi* to obtain serum in order to evaluate the ability of antibodies to neutralize *T. cruzi* infection of cardiomyocytes as described [32]. Green fluorescent protein(GFP)-expressing trypomastigotes, obtained as described previously [27], resuspended in phenol red-free DMEM were preincubated with the same aliquot of sera from immunized or control mice for 30 min at 37°C and parasites were then exposed in triplicate to cardiomyocyte monolayers in Lab Tech chambers at a ratio of 10 parasites/cell. At the end of the incubation period, unbound parasites were removed, complete fresh medium was added to the co-cultures, and parasite multiplication within cell monolayers at 72 h was determined fluorometrically as relative fluorescence units (RFU), as previously described [27], [32]. To microscopically visualize the effect of neutralizing antibodies on cellular infection, we also exposed GFP-expressing trypomastigotes to antibodies from immunized and control mice sera, as described above, and the parasites were then exposed to cardiomyocytes at a parasite-to-cell ratio of 10∶1 for 72 h, as described previously [27], [32]. Cells were fixed and stained with 4′,6-diamidino-2-phenylindole (DAPI) and Alexa Fluor 546 phalloidin for fluorescence confocal microscopy evaluation of infection [26], [33].

### Ethics statement

Animal experiments were performed in strict accordance set forth in the Guide to the Care and Use of Laboratory Animals, DHEW Publication No. (NIH) 78-23 (Revised, 1985). The University of Alabama at Birmingham Institutional Animal Use and Care Committee approved the use of mice as described herein under the approved protocol number 13120997. Animal studies that were conducted at Meharry Medical College were conducted under the National Institutes of Health guidelines on the humane use and care of laboratory animals for biomedical research (Guide for the Care and Use of Laboratory Animals. NIH Publication No. 85-23. Revised 1985). Protocols were approved by the Meharry Medical College Institutional Animal Care and Use Committee.

### Statistical analyses

All experiments were repeated three times. Descriptive statistics, such as means and standard deviations, or standard error of the mean, were computed to study variables of interest. Statistical analyses were performed with paired two-tailed Student t-test or nonpaired two-tailed Student t-test, assuming unequal variance. Statistical significance was defined as *P*<0.05.

## Results

### Generation and characterization of the modified Ad5 vector

**Figure 1:**
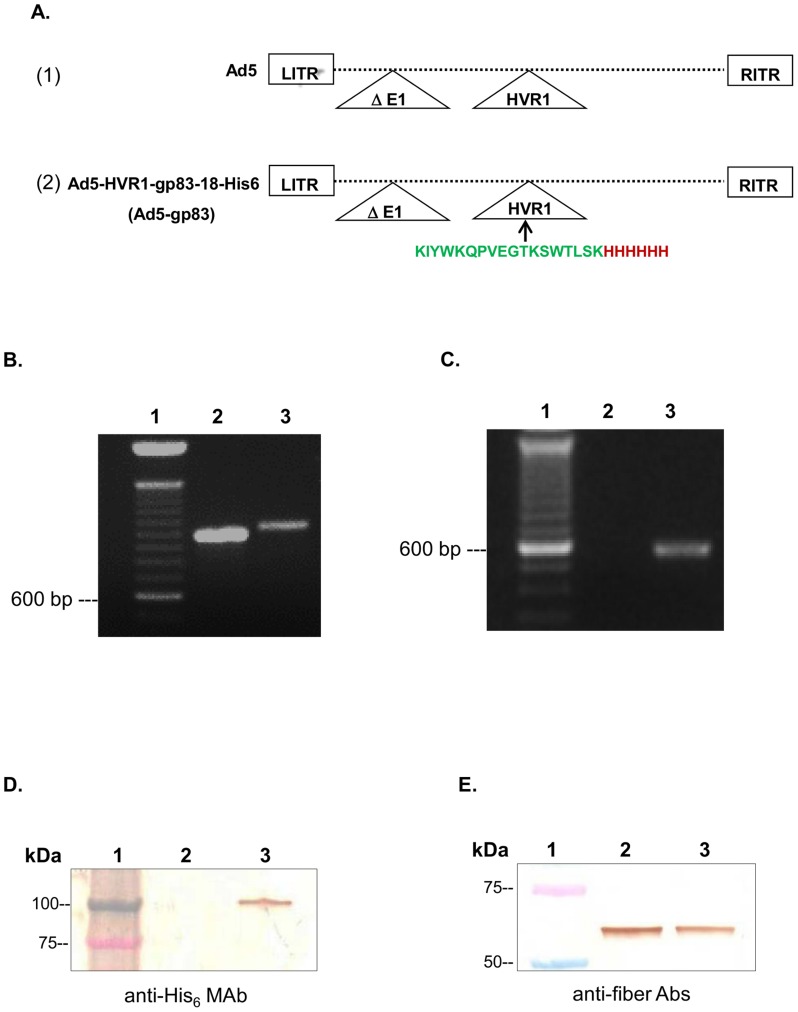
*T. cruzi* gp83 epitope and His_6_ epitope genetically incorporated into the HVR1 of Ad5. Rescued vectors were upscaled and viral DNA was isolated and analyzed to confirm the modification of the relevant genes. A) Schematic of adenoviral genomes. (1) Ad5, a replication-defective adenovirus with unmodified hexon. (2) Ad5-gp83, Ad5 replication-defective genome containing the hexon modification. B) Hexon-specific PCR primers confirmed the presence of the hexon gene in all of the vectors. Lane 1, DNA ladder, lane 2, Ad5; and lane 3, Ad5-gp83. C) *T. cruzi*-His_6_-specific primers confirmed the incorporation of gp83 and His_6_ epitopes. Lane 1, DNA ladder, lane 2, Ad5; and lane 3, Ad5-gp83. D) Western blot analysis confirmed the presence of His_6_ incorporation within the modified vector. E) Western blot analysis confirmed the presence of fiber within the modified vector. In these assays, protein marker (lane 1), Ad5 (lane 2), and Ad5-gp83 (lane 3) were separated by 4–15% SDS-PAGE. The proteins were transferred to polyvinylidene fluoride (PVDF) membranes then incubated with either monoclonal antibodies to His_6_ or fiber. The binding was detected with a HRP-conjugated secondary antibody.

Ad5 (control) and Ad5 vector containing the gp83 mouse MAb 4A4 antibody binding site (GenBank accession #: AY513728.1) (Ad HVR1-gp83-18, from this point forward, will be referred to as Ad5-gp83) as shown in the linear schematic model in Fig. 1A (2) were rescued and upscaled as previously described in [30]. Physical titers and infectious titers were determined to test the stability of the vectors. Based on those two assays, a viral particle/infectious particle (VP/IP) ratio was determined. A normal VP/IP ratio of unmodified Ad ranges from ∼10–30 [30]. We observed an increase in VP/IP ratios for Ad5-gp83 (ratio of 60) when compared to the Ad5 vector (ratio of 30) (Table 1). Based on these observations, the insertion of gp83-His_6_ epitope had minimal effect on the vector's stability.

**Table 1:** Virological properties of vectors used in this study.

Ad5 Vectors	Viral Particles (VP)	Infectious Particles (IP)	VP/IP
**Ad5**	2.38×10^12^ VP/ml	7.9×10^10^ IP/ml	30
**Ad5-HVR1-gp83-18 (Ad5-gp83)**	1.5×10^12^ VP/ml	2.5×10^10^ IP/ml	60

To confirm the antigen capsid-incorporation at the genomic level, PCR analysis was performed using genomic DNA from the purified virions. With respect to hexon-specific PCR, primers were designed to amplify a region upstream of HVR1 and downstream the HVR1 insertion site. Ad5 was found to have a wild type hexon producing a 982 base pairs (bp) fragment (Fig. 1B lane 2). The Ad5-gp83 construct revealed a 1036 bp fragment, suggesting the expected insertion (Fig. 1B lane 3). For the gp83-His_6_-specific PCR, primers were designed to amplify the specific region of the *T. cruzi* antigen-His_6_ insertion. As expected, the PCR for Ad5 did not produce a fragment (Fig. 1C lane 2), while Ad5-gp83 produced a 563 bp fragment indicating the incorporation of the gp83 epitope-His_6_ DNA within the hexon region (Fig. 1C lane 3).

After successful incorporation of the *T. cruzi* antigen gene, we next sought to verify expression of our incorporation at the protein level by Western blot analysis (Fig. 1D). In order to determine if the hexon modified vector displayed the *T. cruzi* gp83 epitope within the hexon region, Ad5 and Ad5-gp83 were subjected to Western blot analysis with His_6_ antibody. The His_6_ protein was detected as a 117 kDa protein band associated with Ad5-gp83 particles (Fig. 1D lane 3). The size of the 117 kDa band corresponds to the expected size of the Ad5 hexon protein with *T. cruzi*-His_6_ epitopes genetically incorporated into the HVR1. There was no His_6_ protein detected on Ad5 wild type particles (Fig. 1D lane 2). The data indicate that the *T. cruzi* antigen and His_6_ were expressed on the hexon gene.

As a control experiment, we sought to determine if the addition of the gp83 epitope onto the Ad5 capsid would dramatically affect the expression of any other capsid proteins. In brief, purified Ad5 and Ad5-gp83 were subjected to Western blot analysis with antibody to fiber protein. The fiber protein was detected as a monomer 64 kDa band associated with both vectors. Notably, the relative fiber protein expression levels for Ad5-gp83 vector were comparable to that of the control vector (Fig. 1E).

### *T. cruzi* antigen capsid-incorporation was exposed on the surface of the virion

**Figure 2:**
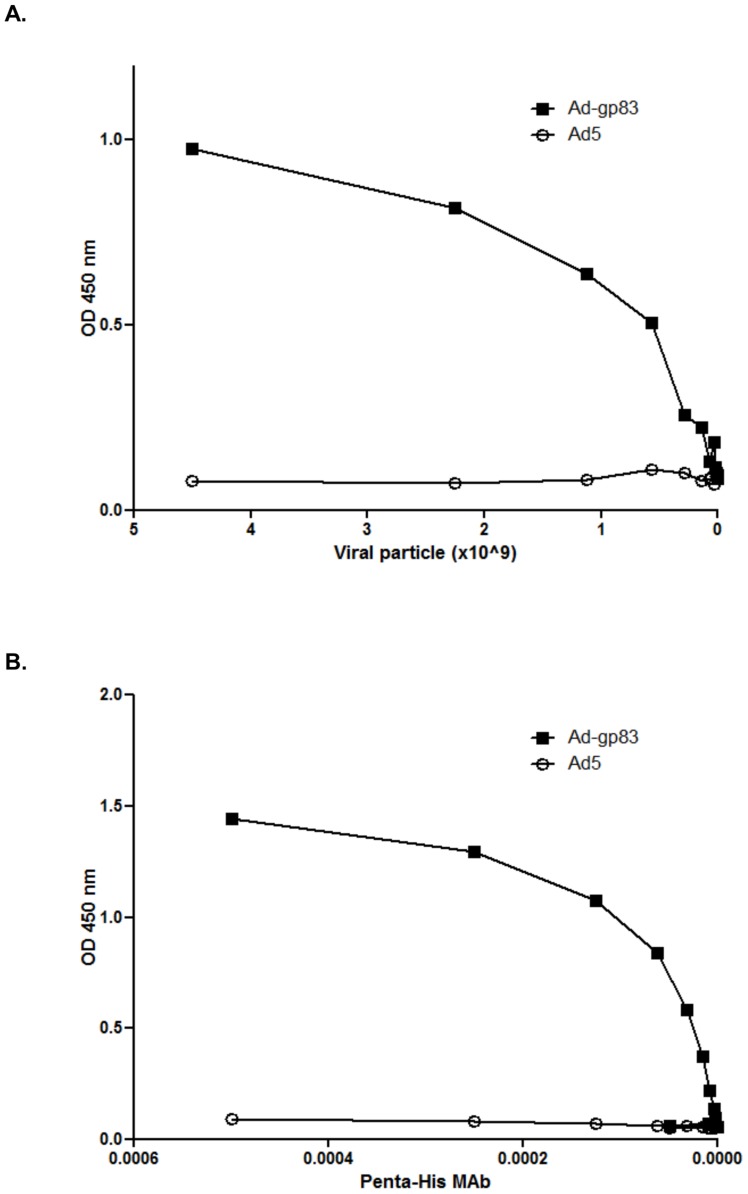
Antigens are exposed on the virion surface. A) Varying amounts (starting at 4.5×10^9^ VP/mouse) of Ad5 or Ad5-gp83 were immobilized into the wells of ELISA plates and incubated with His_6_ MAb. B) Either Ad5 or Ad5-gp83 at 6×10^8^ VP of were immobilized on an ELISA plate followed by varying dilutions of monoclonal antibody to His_6_ (starting at 1∶2,000 dilution). The binding was detected with a HRP-conjugated secondary antibody. OD was read at 450 nm with a microplate reader.

We performed an ELISA assay to verify that the *T. cruzi* antigen and His_6_ were accessible on the virion surface (Fig. 2). Serial dilutions of purified virus were immobilized into the wells of an ELISA plate and incubated with His_6_ MAb. The results showed substantial binding of the His_6_ antibody to the Ad5-gp83, whereas no binding was seen in response to Ad5 control (Fig. 2A).

In order to determine the ability of the His_6_ antibody to bind to capsid-incorporated antigen in a dose-dependent manner a dose-response ELISA assay was performed with His_6_ MAb. A single concentration of Ad5 or Ad5-gp83 was applied to the ELISA plate, followed by the addition of serial dilutions of His_6_ MAb. As predicted, the His_6_ antibody bound to Ad5-gp83 in a dose dependent manner (Fig. 2B). These results indicate that the *T. cruzi* antigen and His_6_ epitope were properly exposed on the virion surface.

### Anti-*T. cruzi* antibodies were produced in mice after immunization with modified vector

**Figure 3:**
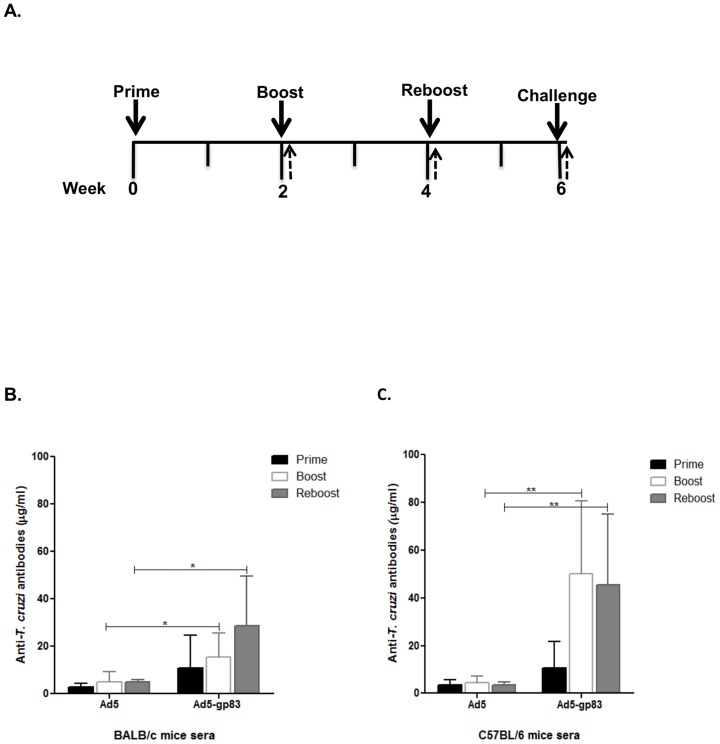
Antigen capsid-incorporation vector elicits an *in vivo T. cruzi* humoral immune response. BALB/c and C57BL/6 mice (n = 7) were primed, boosted, and reboosted with 1×10^10^ VP of Ad vectors. A) Immunization timeline showing when immunizations were performed (solid arrows) and sera was collected (dashed arrows). B) Post-prime, post-boost, and post-reboost BALB/c serum or C) Post-prime, post-boost, and post-reboost C57BL/6 serum was collected for ELISA binding assays. Ten µM of purified gp83 (KIYWKQPVEGTKSWTLSK) antigenic peptide was bound to ELISA plates. The plates were then incubated with serial diluted concentrations of immunized mice serum and the binding antibodies were detected with HRP conjugated secondary antibody. The amount of anti-gp83 antibodies in the sera was calculated based on a standard curve of mouse IgG protein. The values are expressed as the mean ± standard deviation. (*) = *P*≤0.05, and (**) = *P*≤0.01.

To measure *T. cruzi*-specific antibody responses elicited by the hexon-modified Ad5 vector, BALB/c and C57BL/6 mice were immunized with Ad5-gp83, according to the immunization schedule depicted in Fig. 3A. For comparison, mice were immunized with the same dose of Ad5 using the same immunization regimen. Sera were tested for antibodies against *T. cruzi* by ELISA (Fig. 3B and C). Anti-*T. cruzi* IgG levels increased significantly after boost (*P*≤0.05) and reboost (*P*≤0.05) in BALB/c mice immunized with the Ad5-gp83 compared to that of the control (Fig. 3B). There was no significant difference in immunoglobulin levels when comparing prime, boost and reboost in the Ad5-gp83 immunized BALB/c mice group. C57BL/6 mice immunized with the Ad5-gp83 vector as compared to Ad5 controls also showed a significant increase at boost (*P*≤0.01) and reboost (*P*≤0.01). There was a significant increase in immunoglobulin levels when comparing prime and boost (*P*≤0.05) as well as prime and reboost (*P*≤0.05) in the Ad5-gp83 immunized C57BL/6 mice group (statistical analyses not shown on graph). The antibody response at boost and reboost in the C57BL/6 mice were about 30% higher than that of the Ad5-gp83 BALB/C immunized mice (Fig. 3C).

**Figure 4:**
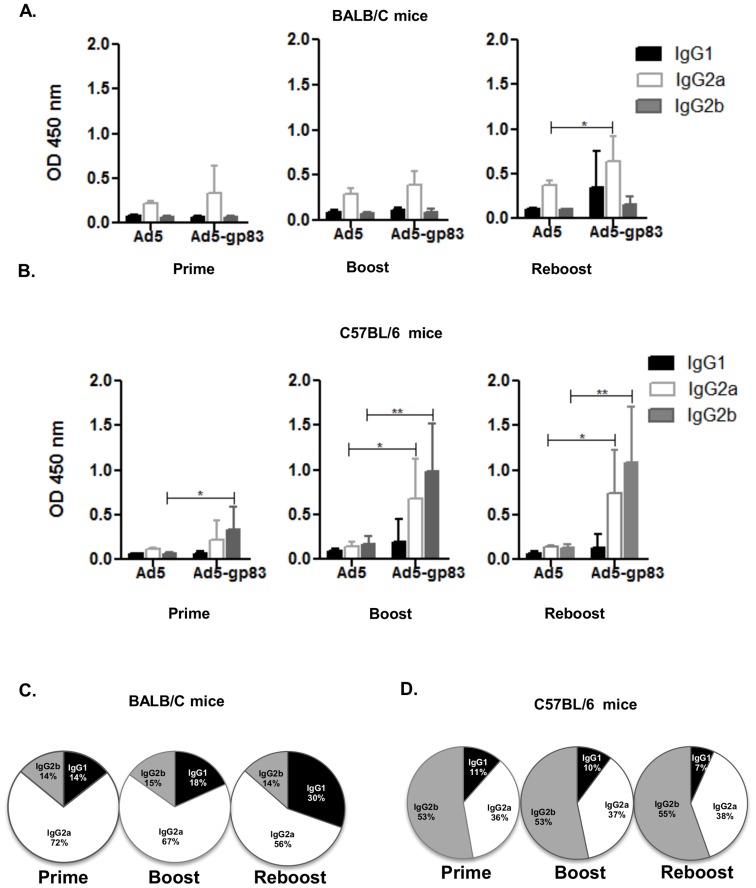
Antigen capsid-incorporation vector elicits an *in vivo T. cruzi* isotype-specific response. Post-prime, post-boost, and post-reboost of BALB/c and C57BL/6 mice (n = 7) serum was used for the isotype-specific assays. A–B) Ten µM of purified gp83 (KIYWKQPVEGTKSWTLSK) antigenic peptide was bound to ELISA plates. Residual unbound peptide was washed from the plates. The plates were then incubated with immunized mice serum followed by isotype-specific antibodies. The binding was detected with HRP conjugated secondary antibodies. OD at 450 nm represents isotype-specific *T. cruzi* gp83 antibody levels in sera. The values are expressed as the mean ± standard deviation. (*) = *P*≤0.05, and (**) = *P*≤0.01. C–D) Pie charts illustrating the isotype distribution patterns post-prime, post-boost, and post-reboost for immunized BALB/c mice or C57BL/6 sera.

Levels of *T. cruzi* IgG subclasses, IgG1, IgG2a, and IgG2b, were assessed to determine the isotype of the humoral immune response being stimulated (Fig. 4). *T. cruzi*-specific peptide was bound to the ELISA plates which were then incubated with immunized mice sera, followed by isotype-specific antibodies, IgG1, IgG2a and IgG2b. Our vaccine strategy might also induce cell-mediated immune response based on the results of IgG subtypes obtained since the Th_1_ IFN-γ cytokine induces class switch to Ig2a, the Treg TGF-β cytokine induces class switch to IgG2b, and Th_2_ cytokine IL-4 induces class switch to IgG1 in mice. It appears that the predominant cell-mediated immune responses are mediated by T helper and Treg cells in our murine model.

The sera from the BALB/c Ad5-gp83 immunized mice had detectable levels of IgG1, IgG2a, and IgG2b levels whereas IgG2a levels shows a significant increase at reboost compared to the control (*P*≤0.05) (Fig. 4A). The IgG1 and IgG2b levels stayed consistently low, although the data shows a slight increase in IgG1 at reboost but the increase was not significant. There was a significant increase in IgG1 levels when comparing prime and boost (*P*≤0.05), as well as a significant increase of IgG2b levels (*P*≤0.05) between prime and reboost in the Ad5-gp83 immunized BALB/c mice group (statistical analyses are not shown on graph). The sera from the Ad5-gp83 immunized BALB/c mice showed that IgG2a was the more dominant isotype (Fig. 4C). The sera from the Ad5-gp83 C57BL/6 immunized mice also had detectable levels of IgG1, IgG2a, and IgG2b (Fig. 4B). There was a significant increase of IgG2a levels at boost (*P*≤0.05) and reboost (*P*≤0.05) compared to that of the Ad5 control. Likewise, IgG2b levels showed a significant increase at prime (*P*≤0.05), boost (*P*≤0.01), as well as reboost (*P*≤0.01) compared to that of the Ad5 control. But surprisingly while IgG2a was the dominant isotype in the BALB/c Ad5-gp83 immunized mice, IgG2b was more dominant in the C57BL/6 Ad5-gp83 immunized mice (Fig. 4D). There was a significant increase in IgG2a levels between prime and boost (*P*≤0.05) and prime and reboost (*P*≤0.05) in the Ad5-gp83 C57BL/6 immunized mice group. There was also a significant increase in IgG2b levels between prime and boost (*P*≤0.05) as well as prime and reboost (*P*≤0.05) in the Ad5-gp83 C57BL/6 immunized mice group (statistical analyses are not shown on graph). The fact that we do not see an apparent difference between boost and reboost responses may suggest that in this model we may only need to give a boost immunization. The IgG subtype response obtained in immunized mice suggests Th_1_, Th_2_ and Treg-type responses.

### Modified Ad5 vector contributed in protection from *T. cruzi* infection

Based on the higher immune response from the C57BL/6 mice, C57BL/6 mice were immunized with the either Ad5 or Ad5-gp83, according to the same immunization schedule depicted in Fig. 3. Two weeks after reboost the mice were injected with a lethal dose of blood trypomastigotes.

**Figure 5:**
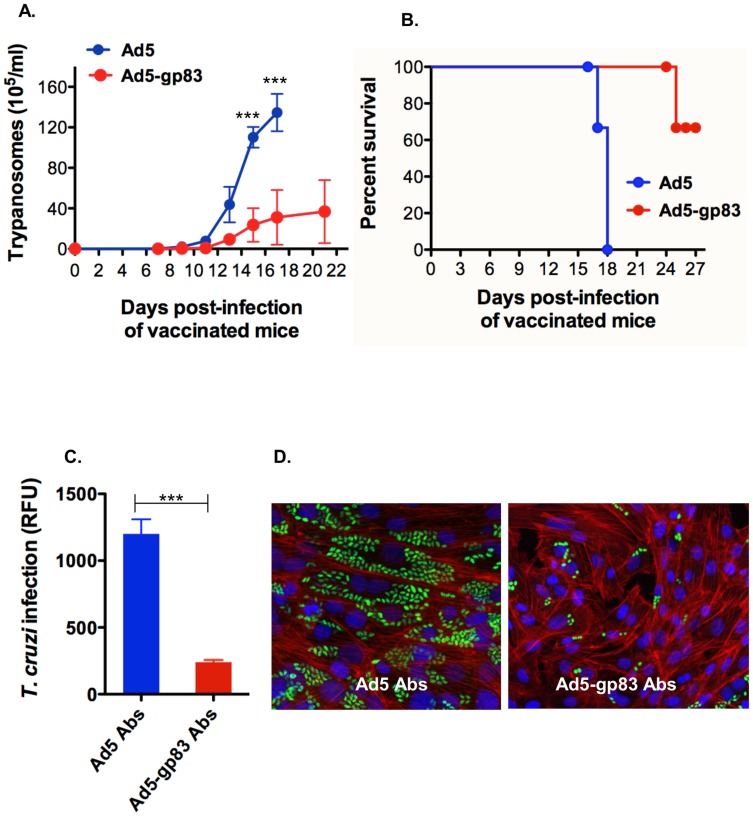
Mice immunization with antigen capsid-incorporation vector protects against the challenge with *T. cruzi* and elicit neutralizing antibodies. A) Parasitemia of vaccinated mice. C57BL/6 mice (5 per group, 6-week old) that were immunized with either Ad5 or Ad5-p83 were challenged intraperitoneally with a lethal dose of blood trypomastigotes (5×10^3^) and the kinetics of parasitemia was determined in 5 µl of blood tail. Data represent the mean values ± SEM. B) Kaplan-Meier survival plot. C) Neutralization of *T. cruzi* infection of cardiomyocytes with Abs from vaccinated mice. Parasite multiplication within cell monolayers was estimated by determining the fluorescence level of parasites expressing green fluorescent protein, which is indicated as relative fluorescence units (RFU) at 72 hours of infection. Data represent the mean values ± SEM of the results from triplicate samples. (***) = *P*≤0.001. D) Fluorescence microscopic observation of the effect of neutralizing antibodies on cardiomyocyte infection by *T. cruzi*. Trypomastigotes expressing GFP were pre-treated with Abs and exposed to cardiomyocytes for 72 h as described in Material and Methods. Abs were obtained from vaccinated mice with either Ad or Ad5-gp83 before *T. cruzi* challenge. GFP-expressing amastigotes are seen inside host cells, host cell nuclei are stained blue, and cellular actin filaments are stained red.

We observed that mice immunized with vector Ad5-gp83 and challenged with a lethal dose of *T. cruzi* blood trypomastigotes presented a significant reduction in the level of parasitemia with respect to the mice control group that received Ad5 vector alone (Fig. 5A). We also observed that there was an increase in the survival rate of mice immunized with Ad5-gp83 with respect to the control group of mice that received Ad5 (Fig. 5B). Anti-gp83 antibodies present in sera obtained from mice immunized with Ad5-gp83 before *T. cruzi* challenge were able to neutralize *T. cruzi* infection of cardiomyocytes *in vitro* with respect to sera obtained from mice immunized with Ad5 vector alone control (Fig. 5C). Fig. 5D shows a fluorescence microscopic observation of the neutralization of *T. cruzi* cellular infection resulting from the treatment of trypomastigotes with sera containing anti-gp83 antibodies from immunized mice with Ad5-gp83 with respect to sera from the control group of mice receiving Ad5. These results indicate that mice immunization with vector Ad5-gp83 confers strong immunoprotection as evident by the significant reduction in the level of parasitemia, increased survival rate and induction of neutralizing antibodies as compared to vaccine control mice.

## Discussion

A safe and effective vaccine against *T. cruzi* has long been in demand, but is not available thus far. Efforts toward vaccine development against *T. cruzi* have been primarily focused on antigens that are expressed on the plasma membrane of the parasite, attached by a glycosylphosphatidylinositol (GPI) anchor [34]–[38]. The surface coat of the invasive trypomastigote form of *T. cruzi* contains trans-sialidases (TS), TS like molecules, mucins and other membrane proteins (reviewed in [34]). In the trypomastigote stage of *T. cruzi*, TS and TS like molecules are glycosylphosphatidylinositol-anchored non-integral membrane proteins that are crucial during the parasite's life cycle and for survival in the mammalian host [35]. A TS-like molecule which is commonly referred to as gp83 was selected for incorporation into our vaccine vector because we have demonstrated that blocking *T. cruzi* with anti-gp83 antibodies reduces trypanosome binding and invasion [36]. The gp83 is only expressed in invasive trypomastigotes but not on non-invasive epimastigotes [23]. Furthermore, our results indicate that the epitope recognized by the neutralizing antibody 4A4 on the gp83 of the trypomastigote is required for binding to phagocytic and non-phagocytic cells to trigger cellular infection [23]. The release of gp83 from trypomastigotes acts on cells to enhance cellular infection [37], [38]. Passive immunization of mice with monovalent Fab fragments of the MAb 4A4 confer strong protection and survival of mice challenged with a lethal dose of trypomastigotes [23]. In addition, we have extensively mapped the gp83 conformational epitope recognized by the neutralizing MAb 4A4 (Pratap *et al.*, 2014 in preparation).

In this study, we examine the humoral responses to the *T-cruzi* gp83-specific antigen that is capsid-incorporated on Ad5 vectors. We generated a recombinant Ad5 vector with an epitope derived from *T. cruzi* gp83 incorporated into the HVR1 region of the major capsid protein hexon. We describe the construction and *in vitro* characterization of Ad5-gp83. The insertion of the *T. cruzi* epitope did not dramatically affect major capsid proteins, such as fiber, or the overall fitness of the vector. The modified vector had normal growth characteristics similar to those of the wild type Ad5. The insertion of the *T. cruzi* epitope was surface exposed via whole-virus ELISA assay demonstration. Importantly, our study provides evidence that immunization with the *T. cruzi* capsid-modified vector elicits a robust humoral antibody response in two different strains of mice and reduces experimental *T. cruzi* infection in C57BL/6 mice.

Generation of different IgG subclasses have been indicated in antibody responses [39]–[42] as well as CD8 responses [39], [43]–[46] after *T. cruzi* infection. In the present study, analysis of the IgG subclasses from the mice immunized with Ad5-gp83 indicated IgG2a as being the dominant response in immunized BALB/c mice (at post-boost and post-reboost time points) and IgG2b the dominant response in immunized C57BL/6 mice (at post-prime, post-boost, and post-reboost time points). In addition to eliciting neutralizing antibodies, the strong IgG2b response induced by the Ad5-gp83 vaccine may also facilitate antibody-dependent cellular cytotoxicity (ADCC) and complement-dependent cytotoxicity (CDC), which are known antibody effector functions of IgG2b. Further study is required to determine if the antigen capsid-incorporation strategy is as effective at stimulating *T. cruzi*-specific cellular immunity as it has been shown here to stimulate humoral immunity.

Earlier works have applied trans-sialidase (TS) antigens for vaccine development against *T. cruzi* [47]–[51] and mucins [52]. Wizel *et al.* was the first to show inhibition of *T. cruzi* infection by using plasmid DNA of TS as a vaccine strategy. However, their findings only focused on the CD+8 T cell response [47]. Vasconcelos *et al.* used different combinations of a plasmid containing the gene encoding the catalytic domain of TS and the recombinant TS protein. While this study confirmed the CD+8 T cell-mediated immune response in BALB/c and C57BL/6 mice, none of the different combinations could protect highly susceptible mice (A/Sn) from *T. cruzi* infection [53]. Based on the promising findings of viral vectors used for vaccine therapy, other groups have chosen to use Ad as a vaccine vector and expressed TS and other *T. cruzi* proteins as a transgene [54]–[58]. Amastigote surface protein-2 (ASP-2) has also been a promising target for vaccine development against Chagas disease [59]–[61]. Our impending approach is to utilize the antigen capsid-incorporation strategy and develop Ad5 vectors that present ASP-2 epitopes to elicit a robust epitope-specific response. To date, the studies in our manuscript herein demonstrate the first time that the antigen capsid-incorporation strategy has been applied for a vaccine development against a *T. cruzi* parasite.

A major obstacle in using Ad5 for vaccine therapy is that the majority of the population has pre-existing immunity (PEI) resulting from natural exposure to the common cold [62]–[65]. The antigen capsid-incorporation strategy to some degree can circumvent PEI in BALB/c mice relative to boost and reboost (Fig. 3B). One of our future strategies to circumvent the PEI is to develop a chimeric Ad5 vector by replacing the entire Ad5 hexon with the hexon from Ad serotype 3. These chimeric vectors will either present and/or express *T. cruzi* antigens. This strategy will allow the evasion of Ad5 immunity, in addition; the dual antigen presentation could theoretically allow a more comprehensive vaccine effort which could potentially control *T. cruzi* infection via the humoral immune response and disease progression via the cellular immune response. Another future strategy to circumvent vector-mediated PEI is to develop *T. cruzi* vaccine vectors from rare adenovirus serotypes (e.g, Ad3, Ad35 or Ad36) [66]–[68].

A protein BLAST search using blastp on the UniProt database (http://www.uniprot.org/) and a tblastn for bioinformatics analysis of the *T. cruzi* genome databases of currently sequenced strains in the NCBI genomes and refseq databases (http://www.ncbi.nlm.nih.gov/refseq) revealed highly significant identity between the epitope recognized by MAb 4A4 and many surface proteins from multiple geographically diverse *T. cruzi* strains typically present in Brazil, Argentina, Chile, Venezuela, Bolivia, Colombia and Mexico (Pratap *et. al.*, 2014 manuscript in preparation) [69], [70]. Accordingly, the identified strains with highly similar protein and nucleotide matches to the MAb 4A4 epitope are CL Brener, Y, Sylvio and Marinkellei (Brazil), CA1 (Argentina), Dm28C (Venezuela), Tulahuen (Chile), SO34 cL4 (Bolivia), Colombiana (Colombia) and MINOA (Mexico). These results suggest that vaccine development with the neutralizing MAb 4A4 epitope delivered by Ad5-gp83 could have a broad spectrum protective effect on *T. cruzi* strains differing in levels of drug resistance and a wide range of geographical coverage; thus being relevant as an important component in the development of a universal vaccine against *T. cruzi*.

In summary, we demonstrated that Ad5-gp83 would be useful in the development of vaccines against Chagas disease, due to the ability of the vector in triggering robust humoral immune responses to a *T. cruzi* antigen and in providing immune-protection against *T. cruzi* challenge by eliciting neutralizing antibodies. The antigen capsid-incorporation strategy is attractive for a complex parasite such as *T. cruzi*, because the adenovirus capsid is extremely amenable to the incorporations of multiple linear and discontinuous epitopes that represent parasite antigens at various life cycles. Therefore, we suggest that this strategy can be manipulated to introduce *T. cruzi* epitopes of immunological importance to induce a robust anti-*T. cruzi* humoral and cellular response. Furthermore, our current study can be viewed as a platform to introduce an effective vaccine methodology for other infectious diseases.
